# The role of allergic history in patients having supratentorial gliomas

**DOI:** 10.1186/2045-7022-1-S1-P103

**Published:** 2011-08-12

**Authors:** Olga Markova, Anna Shmeliova, Oleksander Glavatsky

**Affiliations:** 1 SI The Academician A.P. Romodanov Institute of Neurosurgery under the AMS of Ukraine, Department of Neuroimmunology, Kyiv, Ukraine

## 

Allergization mirrors some peculiarities of the cytokine balance in the human immune system. Individual specific features of immunoreactive patients with gliomas exert effect on the results of their combined treatment, however the role of patient allergization in the clinical course and postoperative treatment still remains unclear.

The goal was to analyze the treatment results of patients with supratentorial gliomas, who had allergic manifestations (to food and some drugs). Comparative analysis of the treatment results was performed for 45 patients with supratentorial (mostly frontal) gliomas, who received a combined treatment (surgery, chemotherapy etc). Tumor histostructure was studied: typical gliomas - 12 cases; anaplastic gliomas - 26 cases; glioblastomas - 7 cases. Two groups of patients were isolated:group I – patients with allergic reactions; group II – patients with uncompromised allergic history. Allergic reactions (allergy to food, etc) were compared to atopic diseases, postoperative complications and duration of the relapse-free disease period.

The study showed that 8 of 45 patients complained of allergic reactions (group I). Specific feature of the tumor histostructure in this group was pronounced lymphoid infiltration, whose intensity correlated with a degree of the allergic history compromise but not with that of glioma anaplasia. The tumor histostructure in group II had some isolated lymphoid infiltrates, mostly in patients with III- IV grade gliomas.

Atopic diseases (asthmatic bronchitis) took place in 1 of 8 allergen-challenged patients. Prior to surgical intervention, in 3 of 8 patients the specific weight of eosinophils exceeded 5%. In half of the allergen-challenged patients (4 of 8) there were the postoperative complications (meningocephalitis – 1 case; bilateral bronchopneumonia – 1 case; laryngotracheitis – 1 case; fever of unrevealed genesis – 1 case). Clinical specificities showed that patients with pronounced glioma lymphoid infiltration had a longer relapse-free period. Further studies may clear up the role of allergic mechanisms not only in gliomogenesis, combined treatment, but also in prognostication of such a treatment.

